# 2-(4-Methyl­phen­oxy)acetohydrazide

**DOI:** 10.1107/S1600536810051937

**Published:** 2010-12-18

**Authors:** Hoong-Kun Fun, Ching Kheng Quah, Shridhar Malladi, Vijesh A. M., Arun M. Isloor

**Affiliations:** a X-ray Crystallography Unit, School of Physics, Universiti Sains Malaysia, 11800 USM, Penang, Malaysia; b Organic Chemistry Division, Department of Chemistry, National Institute of Technology-Karnataka, Surathkal, Mangalore 575 025, India

## Abstract

In the title compound, C_9_H_12_N_2_O_2_, the acetohydrazide group is approximately planar [maximum deviation = 0.034 (2) Å]. In the crystal, mol­ecules are linked *via* inter­molecular N—H⋯O, N—H⋯N and C—H⋯O hydrogen bonds into infinite two-dimensional networks parallel to (001).

## Related literature

For general background to and the biological activity of hydrazide derivatives, see: Isloor *et al.* (2009 ▶); Holla & Udupa (1992 ▶); Ozdemir *et al.* (2009 ▶); Khattab (2005 ▶); Yale *et al.* (1953 ▶). For the preparation of title compound, see: Conti (1964 ▶). For bond-length data, see: Allen *et al.* (1987 ▶). For related structures, see: Fun *et al.* (2009 ▶, 2010*a* ▶,*b* ▶).
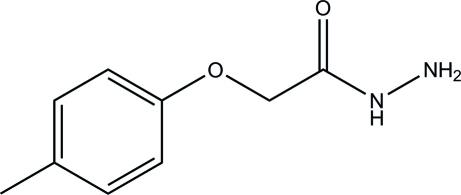


## Experimental

### 

#### Crystal data

C_9_H_12_N_2_O_2_
*M* *_r_* = 180.21
Monoclinic, 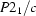

*a* = 6.3833 (2) Å
*b* = 4.0755 (1) Å
*c* = 35.9741 (12) Å
β = 90.018 (2)°
*V* = 935.87 (5) Å^3^
*Z* = 4
Mo *K*α radiation
μ = 0.09 mm^−1^
*T* = 296 K
0.46 × 0.33 × 0.10 mm

#### Data collection

Bruker SMART APEXII CCD area-detector diffractometer
Absorption correction: multi-scan (*SADABS*; Bruker, 2009 ▶) *T*_min_ = 0.959, *T*_max_ = 0.991
15060 measured reflections
2150 independent reflections
1747 reflections with *I* > 2σ(*I*)
*R*_int_ = 0.042

#### Refinement

*R*[*F* ^2^ > 2σ(*F* ^2^)] = 0.067
*wR*(*F* ^2^) = 0.149
*S* = 1.14
2150 reflections
131 parameters
H atoms treated by a mixture of independent and constrained refinement
Δρ_max_ = 0.21 e Å^−3^
Δρ_min_ = −0.16 e Å^−3^

### 

Data collection: *APEX2* (Bruker, 2009 ▶); cell refinement: *SAINT* (Bruker, 2009 ▶); data reduction: *SAINT*; program(s) used to solve structure: *SHELXTL* (Sheldrick, 2008 ▶); program(s) used to refine structure: *SHELXTL*; molecular graphics: *SHELXTL*; software used to prepare material for publication: *SHELXTL* and *PLATON* (Spek, 2009 ▶).

## Supplementary Material

Crystal structure: contains datablocks global, I. DOI: 10.1107/S1600536810051937/hb5765sup1.cif

Structure factors: contains datablocks I. DOI: 10.1107/S1600536810051937/hb5765Isup2.hkl

Additional supplementary materials:  crystallographic information; 3D view; checkCIF report

## Figures and Tables

**Table 1:** Hydrogen-bond geometry (Å, °)

*D*—H⋯*A*	*D*—H	H⋯*A*	*D*⋯*A*	*D*—H⋯*A*
N1—H1*N*1⋯N2^i^	0.92 (3)	2.17 (3)	2.982 (3)	147 (2)
N2—H2*N*2⋯O2^ii^	0.90 (3)	2.14 (3)	3.022 (3)	168 (3)
N2—H1*N*2⋯O2^iii^	0.98 (3)	2.47 (3)	3.166 (3)	128 (2)
C1—H1*A*⋯O2^iv^	0.93	2.53	3.410 (3)	157

Symmetry codes: (i) 
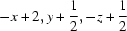
; (ii) 
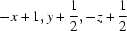
; (iii) 
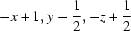
; (iv) 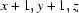.

**Fig. 1.**
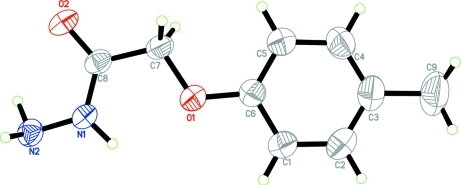
The molecular structure of the title compound showing 50% probability displacement ellipsoids for non-H atoms and the atom-numbering scheme.

**Fig. 2.**
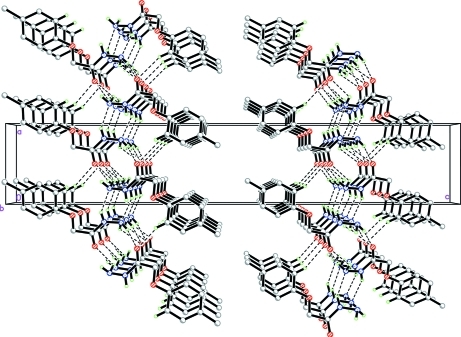
The crystal structure of the title compound, viewed along the b axis. H atoms not involved in hydrogen bonds (dashed lines) have been omitted for clarity.

## supplementary crystallographic information

### Comment

The full therapeutic possibilities of hydrazides were realized after the discovery of isonicotinic acid hydrazide (INH). Hydrazides and their derivatives have been described as useful synthons of various heterocyclic rings (Isloor *et al.*, 2009; Holla & Udupa, 1992). A large number of hydrazides and their derivatives are reported to possess a broad spectrum of biological activities (Ozdemir *et al.*, 2009; Khattab, 2005). The most widely used method to prepare hydrazides is hydrazinolysis of the corresponding esters with hydrazine hydrate (Yale *et al.*, 1953). Prompted by the diverse activities of hydrazides and its derivatives, we have synthesized the title compound to study its crystal structure.

The molecular structure is shown in Fig. 1. The acetohydrazide group (C7/C8/N1/N2/O2) is approximately planar, with the maximum deviation of 0.034 (2) Å at atom C7. Bond lengths and angles are within normal ranges, and comparable to closely related structures (Fun *et al.*, 2009, 2010*a*,*b*). In the solid state (Fig. 2), the molecules are linked *via* intermolecular N2–H1N2···O2, N2–H2N2···O2 and C1–H1A···O2 trifurcated acceptor bonds, together with N1–H1N1···N2 hydrogen bonds, into infinite two-dimensional networks parallel to plane (001).

### Experimental

Ethyl(4-methylphenoxy)acetate  (1.94 g, 0.01 mol) and hydrazine hydrate (99%, 0.02 mol) in ethanol (15 ml) was heated on a water-bath for 6 h. Excess ethanol was removed by distillation. On cooling, colourless needle-shaped crystals of 2-(4-methylphenoxy)acetohydrazide begin to separate. It was collected by filtration and recrystallized from ethanol. Yield: 1.2 g, 67.0 %, *M.p.*: 411-413K. (Conti, 1964).

### Refinement

H1N1, H1N2 and H2N2 were located in a difference Fourier map and allowed to refined freely. The remaining H atoms were positioned geometrically and refined using a riding model with C–H = 0.93 –0.97 Å and *U*_iso_(H) = 1.2 or 1.5 *U*_eq_(C). The highest residual electron density peak is located at 0.94 Å from H9C and the deepest hole is located at 0.94 Å from C8.

### Crystal data

**Table:**

C_9_H_12_N_2_O_2_	*F*(000) = 384
*M**_r_* = 180.21	*D*_x_ = 1.279 Mg m^−^^3^
Monoclinic, *P*2_1_/*c*	Mo *K*α radiation, λ = 0.71073 Å
Hall symbol: -P 2ybc	Cell parameters from 4756 reflections
*a* = 6.3833 (2) Å	θ = 3.2–25.4°
*b* = 4.0755 (1) Å	µ = 0.09 mm^−^^1^
*c* = 35.9741 (12) Å	*T* = 296 K
β = 90.018 (2)°	Block, colourless
*V* = 935.87 (5) Å^3^	0.46 × 0.33 × 0.10 mm
*Z* = 4	

### Data collection

**Table:**

Bruker SMART APEXII CCD area-detector diffractometer	2150 independent reflections
Radiation source: fine-focus sealed tube	1747 reflections with *I* > 2σ(*I*)
graphite	*R*_int_ = 0.042
φ and ω scans	θ_max_ = 27.5°, θ_min_ = 2.3°
Absorption correction: multi-scan (*SADABS*; Bruker, 2009)	*h* = −8→8
*T*_min_ = 0.959, *T*_max_ = 0.991	*k* = −5→5
15060 measured reflections	*l* = −46→45

### Refinement

**Table:**

Refinement on *F*^2^	Primary atom site location: structure-invariant direct methods
Least-squares matrix: full	Secondary atom site location: difference Fourier map
*R*[*F*^2^ > 2σ(*F*^2^)] = 0.067	Hydrogen site location: inferred from neighbouring sites
*wR*(*F*^2^) = 0.149	H atoms treated by a mixture of independent and constrained refinement
*S* = 1.14	*w* = 1/[σ^2^(*F*_o_^2^) + (0.0368*P*)^2^ + 0.8025*P*] where *P* = (*F*_o_^2^ + 2*F*_c_^2^)/3
2150 reflections	(Δ/σ)_max_ = 0.001
131 parameters	Δρ_max_ = 0.21 e Å^−^^3^
0 restraints	Δρ_min_ = −0.16 e Å^−^^3^

### Special details

**Table:**

Geometry. All esds (except the esd in the dihedral angle between two l.s. planes) are estimated using the full covariance matrix. The cell esds are taken into account individually in the estimation of esds in distances, angles and torsion angles; correlations between esds in cell parameters are only used when they are defined by crystal symmetry. An approximate (isotropic) treatment of cell esds is used for estimating esds involving l.s. planes.
Refinement. Refinement of F^2^ against ALL reflections. The weighted R-factor wR and goodness of fit S are based on F^2^, conventional R-factors R are based on F, with F set to zero for negative F^2^. The threshold expression of F^2^ > 2sigma(F^2^) is used only for calculating R-factors(gt) etc. and is not relevant to the choice of reflections for refinement. R-factors based on F^2^ are statistically about twice as large as those based on F, and R- factors based on ALL data will be even larger.

### Fractional atomic coordinates and isotropic or equivalent isotropic displacement parameters (Å^2^)

**Table:**

	*x*	*y*	*z*	*U*_iso_*/*U*_eq_	
O1	0.8895 (2)	0.9310 (5)	0.16293 (4)	0.0486 (5)	
O2	0.5021 (2)	0.4131 (5)	0.20181 (5)	0.0518 (5)	
N1	0.8051 (3)	0.6119 (5)	0.22475 (5)	0.0386 (5)	
N2	0.7875 (3)	0.4400 (6)	0.25893 (6)	0.0424 (5)	
C1	1.1646 (4)	1.1960 (7)	0.13257 (7)	0.0492 (6)	
H1A	1.2259	1.2295	0.1557	0.059*	
C2	1.2626 (4)	1.3059 (7)	0.10089 (8)	0.0568 (7)	
H2A	1.3912	1.4113	0.1030	0.068*	
C3	1.1765 (5)	1.2649 (7)	0.06605 (8)	0.0581 (7)	
C4	0.9852 (5)	1.1122 (8)	0.06415 (7)	0.0614 (8)	
H4A	0.9226	1.0843	0.0410	0.074*	
C5	0.8816 (4)	0.9975 (7)	0.09561 (7)	0.0529 (7)	
H5A	0.7514	0.8967	0.0935	0.063*	
C6	0.9740 (3)	1.0352 (6)	0.12983 (6)	0.0407 (5)	
C7	0.7042 (3)	0.7393 (6)	0.16147 (6)	0.0405 (5)	
H7A	0.7183	0.5731	0.1423	0.049*	
H7B	0.5864	0.8784	0.1550	0.049*	
C8	0.6637 (3)	0.5776 (6)	0.19811 (6)	0.0374 (5)	
C9	1.2895 (6)	1.3842 (10)	0.03148 (9)	0.0890 (11)	
H9A	1.3602	1.2033	0.0199	0.133*	
H9B	1.3900	1.5489	0.0383	0.133*	
H9C	1.1897	1.4761	0.0144	0.133*	
H1N1	0.919 (4)	0.745 (7)	0.2202 (7)	0.058 (8)*	
H2N2	0.712 (5)	0.572 (8)	0.2738 (8)	0.066 (9)*	
H1N2	0.705 (5)	0.243 (9)	0.2537 (8)	0.070 (9)*	

### Atomic displacement parameters (Å^2^)

**Table:**

	*U*^11^	*U*^22^	*U*^33^	*U*^12^	*U*^13^	*U*^23^
O1	0.0452 (9)	0.0592 (11)	0.0415 (9)	−0.0185 (8)	0.0003 (7)	0.0002 (8)
O2	0.0347 (8)	0.0604 (11)	0.0604 (11)	−0.0179 (8)	−0.0005 (7)	0.0015 (9)
N1	0.0297 (9)	0.0437 (11)	0.0424 (10)	−0.0053 (8)	0.0015 (7)	0.0008 (9)
N2	0.0350 (10)	0.0473 (12)	0.0448 (11)	0.0032 (9)	0.0032 (8)	0.0029 (10)
C1	0.0411 (12)	0.0547 (15)	0.0519 (14)	−0.0064 (11)	−0.0038 (10)	0.0032 (12)
C2	0.0443 (13)	0.0590 (17)	0.0671 (18)	−0.0089 (12)	0.0074 (12)	0.0027 (14)
C3	0.0676 (17)	0.0532 (16)	0.0534 (16)	−0.0053 (14)	0.0135 (13)	0.0042 (13)
C4	0.0749 (19)	0.0663 (19)	0.0431 (14)	−0.0118 (16)	−0.0020 (13)	0.0015 (13)
C5	0.0507 (14)	0.0592 (17)	0.0487 (14)	−0.0118 (13)	−0.0026 (11)	−0.0002 (12)
C6	0.0405 (11)	0.0396 (12)	0.0418 (12)	−0.0001 (10)	0.0020 (9)	−0.0010 (10)
C7	0.0329 (11)	0.0430 (13)	0.0455 (13)	−0.0068 (10)	−0.0008 (9)	−0.0037 (10)
C8	0.0283 (10)	0.0385 (11)	0.0453 (12)	0.0015 (9)	0.0027 (8)	−0.0053 (10)
C9	0.107 (3)	0.091 (3)	0.070 (2)	−0.022 (2)	0.0300 (19)	0.010 (2)

### Geometric parameters (Å, °)

**Table:**

O1—C6	1.375 (3)	C3—C4	1.372 (4)
O1—C7	1.418 (3)	C3—C9	1.518 (4)
O2—C8	1.238 (3)	C4—C5	1.392 (4)
N1—C8	1.323 (3)	C4—H4A	0.9300
N1—N2	1.420 (3)	C5—C6	1.374 (3)
N1—H1N1	0.92 (3)	C5—H5A	0.9300
N2—H2N2	0.90 (3)	C7—C8	1.496 (3)
N2—H1N2	0.98 (3)	C7—H7A	0.9700
C1—C2	1.375 (4)	C7—H7B	0.9700
C1—C6	1.385 (3)	C9—H9A	0.9600
C1—H1A	0.9300	C9—H9B	0.9600
C2—C3	1.378 (4)	C9—H9C	0.9600
C2—H2A	0.9300		
			
C6—O1—C7	117.73 (17)	C6—C5—H5A	120.4
C8—N1—N2	121.39 (19)	C4—C5—H5A	120.4
C8—N1—H1N1	118.1 (16)	C5—C6—O1	125.0 (2)
N2—N1—H1N1	120.5 (16)	C5—C6—C1	119.6 (2)
N1—N2—H2N2	105.3 (19)	O1—C6—C1	115.4 (2)
N1—N2—H1N2	106.3 (17)	O1—C7—C8	110.74 (17)
H2N2—N2—H1N2	108 (2)	O1—C7—H7A	109.5
C2—C1—C6	119.7 (2)	C8—C7—H7A	109.5
C2—C1—H1A	120.2	O1—C7—H7B	109.5
C6—C1—H1A	120.2	C8—C7—H7B	109.5
C1—C2—C3	122.2 (2)	H7A—C7—H7B	108.1
C1—C2—H2A	118.9	O2—C8—N1	123.2 (2)
C3—C2—H2A	118.9	O2—C8—C7	118.52 (19)
C4—C3—C2	117.1 (2)	N1—C8—C7	118.25 (19)
C4—C3—C9	121.8 (3)	C3—C9—H9A	109.5
C2—C3—C9	121.1 (3)	C3—C9—H9B	109.5
C3—C4—C5	122.3 (3)	H9A—C9—H9B	109.5
C3—C4—H4A	118.8	C3—C9—H9C	109.5
C5—C4—H4A	118.8	H9A—C9—H9C	109.5
C6—C5—C4	119.2 (2)	H9B—C9—H9C	109.5
			
C6—C1—C2—C3	0.7 (4)	C7—O1—C6—C1	−174.4 (2)
C1—C2—C3—C4	0.9 (5)	C2—C1—C6—C5	−2.3 (4)
C1—C2—C3—C9	−179.1 (3)	C2—C1—C6—O1	179.2 (2)
C2—C3—C4—C5	−0.9 (5)	C6—O1—C7—C8	165.76 (19)
C9—C3—C4—C5	179.1 (3)	N2—N1—C8—O2	4.7 (3)
C3—C4—C5—C6	−0.6 (5)	N2—N1—C8—C7	−173.8 (2)
C4—C5—C6—O1	−179.4 (2)	O1—C7—C8—O2	176.8 (2)
C4—C5—C6—C1	2.2 (4)	O1—C7—C8—N1	−4.6 (3)
C7—O1—C6—C5	7.2 (4)		

### Hydrogen-bond geometry (Å, °)

**Table:**

*D*—H···*A*	*D*—H	H···*A*	*D*···*A*	*D*—H···*A*
N1—H1N1···N2^i^	0.92 (3)	2.17 (3)	2.982 (3)	147 (2)
N2—H2N2···O2^ii^	0.90 (3)	2.14 (3)	3.022 (3)	168 (3)
N2—H1N2···O2^iii^	0.98 (3)	2.47 (3)	3.166 (3)	128 (2)
C1—H1A···O2^iv^	0.93	2.53	3.410 (3)	157.

Symmetry codes: (i) −*x*+2, *y*+1/2, −*z*+1/2; (ii) −*x*+1, *y*+1/2, −*z*+1/2; (iii) −*x*+1, *y*−1/2, −*z*+1/2; (iv) *x*+1, *y*+1, *z*.
